# Supplementary material for: Chromothripsis during telomere crisis is independent of NHEJ, and consistent with a replicative origin
Source: Genome Res. 2019 May;29(5):737–49. doi: 10.1101/gr.240705.118 (PMC6499312; doi:10.1101/gr.240705.118)
Supplement: Supplemental Material [file supp_gr.240705.118_Supplemental_file_1.zip › contigs/annotated_contigs/DB68/contig.2.DB68_length_445_mean_cov_8.08988764045.docx]

**DB68_length_445_mean_cov_8.08988764045**

```
TTTCTCAGGTTCTCAGTACACAGCGGGAAACAAGTCAGTATTCCTGGCCTGGTGGAGCTCAGTCAAGTGGGGAGAAGATAAGCAGACAA
 >chr20:536625-536804 + E=4e-87
ATCTAGCATTGCAAACTGGGATTATCGCCAGGAGAGACAAGTGGGCGGTGTATGAGAGTGCATGAGAGTAGAGCCGACCTAGT|CGAGG
                                                                                    >chr
GC|CAAGCGGGACGGCGGGCCGCCGCCCCCGGGACCCGGGCCGGCCGAGGAGGGGGCGCGCGAGCCCGGCTGGTGCAAGACCCCGAGCG
20:306587-306860 + E=7e-153
GCCACATCAAGAGGCCGATGAACGCATTCATGGTGTGGTCGCAGCACGAACGGCGGAAGATCATGGACCAGTGGCCCGACATGCACAAC

GCCGAGATCTCCAAGCGCCTGGGCCGCCGCTGGCAGCTGCTGCAGGACTCGGAGAAGATCCCGTTCGTGCGGGAGGCGGAGCGGCTGCG

GC
```
